# Lost in the Woods? Place Recognition for Navigation in Difficult Forest Environments

**DOI:** 10.3389/frobt.2020.541770

**Published:** 2020-11-27

**Authors:** James Garforth, Barbara Webb

**Affiliations:** School of Informatics, University of Edinburgh, Edinburgh, United Kingdom

**Keywords:** visual perception, place recognition, forests, scene statistics, navigation, SLAM, field robotics

## Abstract

Forests present one of the most challenging environments for computer vision due to traits, such as complex texture, rapidly changing lighting, and high dynamicity. Loop closure by place recognition is a crucial part of successfully deploying robotic systems to map forests for the purpose of automating conservation. Modern CNN-based place recognition systems like NetVLAD have reported promising results, but the datasets used to train and test them are primarily of urban scenes. In this paper, we investigate how well NetVLAD generalizes to forest environments and find that it out performs state of the art loop closure approaches. Finally, integrating NetVLAD with ORBSLAM2 and evaluating on a novel forest data set, we find that, although suitable locations for loop closure can be identified, the SLAM system is unable to resolve matched places with feature correspondences. We discuss additional considerations to be addressed in future to deal with this challenging problem.

## 1. Introduction

Mobile robotic systems have the potential to aid in forest management by improving the efficiency, scale and accuracy of tree health data gathering. In order to do this, robots must be able to navigate autonomously around large GPS-denied forest environments where modern visual Simultaneous Localization and Mapping (SLAM) techniques often struggle (Garforth and Webb, 2019).

An important part of the SLAM pipeline is loop closure, wherein the system recognizes that it has returned to a previously visited location (or “place”) and can update its beliefs about intervening states, reducing the effect of drifting sensor readings. The place recognition algorithms used for loop closure in many classic SLAM techniques work by converting images to local descriptors and performing a search over previous descriptors for a likely match.

Deep learning approaches, particularly Convolutional Neural Networks (CNNs), such as NetVLAD (Arandjelovic et al., 2016), have demonstrated substantial promise for matching of images or places from databases. Less has been done to investigate their effectiveness at working for robotic loop closure applications. These often require fine place granularity, real-time performance, and the ability to deal with distinctive environments that are not classically represented in the large place datasets, such as forests.

In this paper, we identify NetVLAD as a candidate for improving loop closure in forest environments, and demonstrate its superior performance, compared against a baseline of traditional approaches, in a series of experiments. Our main contributions are as follows:

- Demonstrating the capacity of NetVLAD's underlying architecture for describing forest scenes.
- Comparison with state of the art loop closure techniques to demonstrate state of the art performance on challenging forest datasets, even in the presence of changing environmental conditions.
- Evaluation of NetVLAD as loop closure mechanism for a robot performing SLAM in a forest using a new data set.

## 2. Related Work

### 2.1. Visual SLAM

Visual Simultaneous Localization and Mapping systems are typically feature-based, extracting salient features from video frames in order to track the motion of the camera over time. Since Klein and Murray (2007) this tracking tends to be done in its own thread to improve performance, separated from the less time critical task of building a 3D map of the world by feature triangulation. The influential ORBSLAM improves tracking performance further with fast ORB features, as well as multiple techniques for map optimization.

Other sensors than a single camera have been used for SLAM applications, such as stereo pairs or depth cameras (RGBD, as in Whelan et al., 2015). Both of these provide useful depth information to a SLAM system, but at the cost of power, weight, computational complexity and their own unique challenges. The setup of a stereo camera's baseline determines the range at which it can reliably estimate depth, and it must still be able to match features between its two images. An RGBD camera relies on reflected infrared light for depth estimation, and so is sensitive to ambient lighting or reflective properties of a scene. These problems are less prevalent under controlled laboratory conditions, but in the field many researchers still opt for a monocular camera setup.

Combining camera with inertial data, as in OKVIS (Leutenegger et al., 2015) and VINS-Mono (Qin et al., 2018), helps to improve local motion estimation, but requires carefully co-calibrated sensor data. This data is absent from most existing datasets, limiting a thorough analysis of visual-inertial SLAM performance.

Many recent works in monocular SLAM have revolved around replacing discrete features with aligning images directly through minimizing photometric error. The most successful of these are LSD-SLAM (Engel et al., 2014) and its successor DSO (Engel et al., 2017). These “direct” approaches have the advantage of not needing to detect corners in images and are therefore more robust to scenes containing large featureless regions like walls. Feature extraction also often accounts for much of the processing in feature-based SLAM, so direct systems can achieve performance benefits by forgoing it. These systems still have their limitations, however, and in their comparison of the two approaches, Yang et al. (2018) note that direct methods are more vulnerable to the effect of rolling shutter cameras and to highly dynamic lighting. The abundance of these in available forest data, as well as the lack of necessary photometric calibration, make feature-based methods an easier choice for mapping forests. Supporting the continued use of the feature-based approach, in Gao et al. (2018) the loop-closure enabled version of DSO is compared against ORBSLAM2 and accuracy of the two systems is notably similar.

### 2.2. Navigating and Mapping Forests

Traditionally, work in the field of forest mapping has been done from stationary or ground-based platforms, using heavy and power-hungry laser-based scanning, and requiring offline processing of the data to form a map (Takashi et al., 2014; Pierzchała et al., 2018). Mobile, online systems often extract only tree trunks from the scene, such as in Schultz et al. (2016) or Liao et al. (2016), a simplification that has also been used for RGBD systems in Fan et al. (2020). This avoids much of the complexity of the forest scene but also limits the usefulness of the final map to only information about placement of trees. While recent works show impressive results for improving laser-based mapping of the whole scene (Tinchev et al., 2018), laser-based sensors are still expensive and need a lot of power. A niche remains for navigating in real time using only monocular cameras, with the aim of enabling deployment on light, nimble and low cost Unmanned Aerial Vehicles (UAVs).

A number of recent works have looked at the problem of visual trail following under forest canopy, starting with Giusti et al. (2015) and more recently Maciel-Pearson et al. (2018). These represent only reactive navigation, rather than mapping, but what they do show us is the potential of neural network based approaches for cutting through the visual complexity of forest scenes. Silva et al. (2020) have recently improved such a trail following system by integrating depth information extracted from a SLAM system, LSD-SLAM (Engel et al., 2014), to augment their trail following, but do not evaluate its performance.

Natural environments like forests present a series of problems for traditional visual SLAM systems, as outlined in Garforth and Webb (2019). Firstly, they undergo frequent rapid changes in illumination due to the effect of the canopy blocking parts of the bright sky. Secondly, they contain a lot of in-scene motion as leaves and ground vegetation move with the wind. Finally, all of those plants result in an abundance of texture that can overwhelm feature detection algorithms.

### 2.3. Loop Closure

Visual SLAM systems keep track of the motion of the camera through the world over time, but due to sensor noise and other sources of inaccuracy, this ego-motion estimate experiences some drift. If the system can detect when it has returned to a previously visited location then it can estimate and optimize out drift that accrued in between. This is the purpose of loop closure. While loop closure and place recognition are similar tasks, they have notably different priorities and deployment or evaluation challenges. The “places” in place recognition datasets are sampled from a wide variety of geographic locations, can represent hundreds of square meters of space (e.g., a large city intersection) and do not usually overlap with each other. A proposed match here is clearly correct or incorrect. In a robotic loop closure context, input images are a continuous, if down-sampled, video stream with no discrete places and plenty of overlap. The challenge here is not to always find all of the closest matches for all images, but rather to recognize the general similarity of the current location to a previously visited region, and thus initiate a process of view alignment.

Three state of the art loop closure algorithms are compared in Lowry and Andreasson (2018): a binary Bag of Words (BoW) model similar to that used in ORBSLAM2 (Gálvez-López and Tardós, 2012; Mur-Artal and Tardós, 2017), FAB-MAP (Cummins and Newman, 2008), and a Vectors of Locally Aggregated Descriptors (VLAD, Jégou et al., 2010). All of these methods reduce images to easily comparable descriptors that can be used to judge image similarity. Lowry and Andreasson compare performance of various descriptor sizes, on a number of datasets including one section of forest, and find that VLAD performs best in most cases.

### 2.4. Deep Learning and Place Recognition

Image matching is a popular domain for Deep Neural Networks, and when the images being matched against represent locations the task is known as place recognition (Lowry et al., 2015). In many areas of computer vision, DNNs have led to rapid advancement in image processing performance, demonstrating robustness to appearance (Gomez-Ojeda et al., 2015), lighting (Gomez-Ojeda et al., 2018), and viewpoint changes (Chen et al., 2017).

In the case of Kendall et al. (2015), Walch et al. (2017), and more recently Li et al. (2019), the output can be as complex as a 6 degree of freedom pose regression respective to the original “map” of images. What all of these systems have in common, however, is that the domain against which they can match novel images is fixed at the point at which they are trained. This makes them unsuitable for deployment in many real world robotic applications, such as loop closure, where the environment has not been previously mapped.

More suitable are networks designed to produce descriptors, similarly to classical approaches. Notable among these is NetVLAD (Arandjelovic et al., 2016), which is trained end-to-end for the place recognition task rather than using representations from a similar task, such as object classification. NetVLAD combines a layer replicating the descriptive power of VLAD descriptors on top of features learned from training on datasets of millions of scene images. The result is a network trained to produce feature vectors where the euclidean distance between two compared vectors should be smaller when the images they were produced from observe the same scene. NetVLAD's descriptors are small enough to be plausibly used for real time place matching, and have been shown to be robust to a lot of visual appearance change.

The CNN-based method of Chen et al. (2017) takes a different approach from whole-image descriptors, instead describing regions within images in a way that makes them robust to partial views of scenes. Vysotska and Stachniss (2019) have taken what could be considered the opposite approach and attempt to match not single image descriptors but sequences of image descriptors. In this way they adaptively define what a “place” is in each context, and are much better prepared to work with recognition in continuous video data. Unfortunately, this work does not have publicly available code, so we focus on the network they built upon: NetVLAD.

## 3. Methods

### 3.1. Datasets

**Figure 1:**
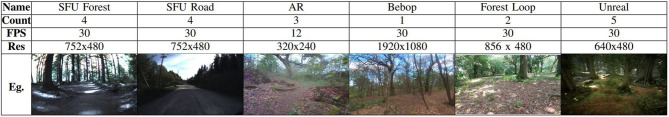
Table showing datasets used in this work, with video count, frame rate, resolution and an example frame.

The forest datasets used in this work were mostly taken from Garforth and Webb (2019), and are summarized in Figure 1.

The SFU Mountain dataset (Bruce et al., 2015) follows the same route along a mountain path on 4 separate occasions under different environmental conditions (dry, wet, dusk, and night) and provides hand-identified matches between a sub sample of places along that route. We split it into “Road” and “Forest” sections based on when the vehicle turns onto a path under forest canopy.

The Hillwood datasets were recorded from two different aerial vehicles: Parrot's AR and Parrot Bebop drones. These represent more difficult forest paths with less of a defined track and less constrained in motion than the ground vehicle in SFU. We supplement these with a new dataset, Forest Loop, also recorded on a Bebop drone. The aim with this dataset was to follow a simple circuit and keep the motion as smooth as possible. In this way it is ideal for testing SLAM's frame to frame tracking as well as loop closure.

Finally, the Unreal dataset follows UAV-like paths similar to Hillwood and Forest Loop, but does so in a photorealistic simulated forest rendered with the Unreal game engine.

### 3.2. Recognizing Forests

Of the versions of NetVLAD reported in Arandjelovic et al. (2016), the most successful builds on VGG-16 (Simonyan and Zisserman, 2014). Before we test NetVLAD on the forest places task, we want to confirm that the underlying network has the ability to classify our datasets as forests. It is the descriptive ability of this network that NetVLAD's final pooling layer builds upon, so without it further tests would be unnecessary.

We use a Keras implementation of VGG-16 by Kalliatakis (2017) to evaluate whether the network can correctly identify forest data. The weights provided for this version of VGG-16 are pre-trained for classification on Places365 (Zhou et al., 2017), which according to the authors provides a “quasi-exhaustive” dataset of possible scene categories including a variety of different types of forest.

Each video frame is resized to a standard input size (224 × 224) and passed through the network, which provides an ordered list of possible scene category labels. The top prediction is used as the classification label for the frame. We record the percentage of frames classified as one of the “forest” labels, as well as noting other commonly assigned labels. This process is repeated for each dataset.

### 3.3. Recognizing Places in Forests

#### 3.3.1. Setup

We set up NetVLAD using the Tensorflow code and pre-trained weights provided by Cieslewski et al. (2018). The only parameter that we have to set is how many of the principal descriptor dimensions to use in our descriptors. As the original authors establish a good trade-off between accuracy and size at 128 dimensions, and we are interested in real time performance, we use that value.

#### 3.3.2. Evaluation

We duplicate the methodology described in Lowry and Andreasson (2018) for evaluating place recognition performance. NetVLAD is used to produce descriptors for each image in the database and each query image. For each query, the squared euclidean distance is calculated between the query descriptor and each of the database descriptors and the top N are considered as potential matches. If one of these matches is within D images of the correct match in the sequence then it is considered to be a true positive. As the datasets tested here are of a small scale compared to city-sized datasets NetVLAD was originally demonstrated on, values of N and D are set to 1 and 2, respectively.

To make this task more difficult, database and query images are also cropped from the full image frame such that they are offset by 40% from one another.

#### 3.3.3. Evaluating on Data With Ground Truth

We use the same two SFU video pairs as Lowry and Andreasson (2018): with the dry conditions as our database set and the wet or dusk conditions as query sets. For fair comparison with the original results we report performance for the whole datasets, but as we are specifically interested in performance under the more challenging canopy covered section, we also report results for a test set with the open road sections removed. Note that in this case the database set is not reduced, as we would expect this to artificially improve performance.

#### 3.3.4. Evaluation on Data With No Ground Truth

We wish to evaluate performance on more forest data than just the SFU videos, but what little data is available does not have ground truth, primarily due to the poor performance of GPS under canopy cover. Instead we use a methodology of splitting a single video into two by alternately sampling frames. This way, our database and query videos do not contain any of the same frames but two frames sampled from next to each other should usually still represent the same place.

There are two parameters to consider when using this method. First, the step size between places in a video, which we set to 3 s (36 or 90 frames for Hillwood AR and Bebop, respectively) in order to generate an equivalent number of places as in the SFU dataset (~150). Given the speed of the UAV recording the Hillwood data, this offset should represent a maximum of 5 m between places. The second parameter is the offset between the database and query frames. At an offset of 0, the sets would be identical, whereas if the offset equals the step size the “ground truth” identity of the frames would be misaligned. We test a range of values between these two unhelpful extremes.

### 3.4. Loop Closure

Our final evaluation is of NetVLAD's potential as a loop closure mechanism in visual SLAM, which we perform in two ways. Firstly, we compare the descriptors generated for each dataset route against themselves and plot the distances as a confusion matrix to look for regions of low distance at the sites of known loop closures. Settings for distance thresholds or other mechanisms of identifying a loop closure will vary between SLAM systems, but usually rely on repeated recognition of similarity within a short period of time. This method allows us to evaluate whether regions of similarity emerge from the system without artificially defining the boundaries of “places” within the data.

Secondly. we perform an integration with ORBSLAM2 (Mur-Artal et al., 2015), a popular visual SLAM system, which uses DBoW2 (Gálvez-López and Tardós, 2012), a Bag of Words method, to propose potential loop closures. We replaced DBoW2 with a simple threshold on the distance between NetVLAD descriptors to decide when to propose an image as a loop closure. Note at this stage the test system is not optimized for running in real time and so the image descriptors are pre-calculated for the whole dataset, and loaded from a file for comparison at runtime. ORBSLAM2 evaluates our loop closures as usual, extracting features from new and old images to try and align them.

This system needed a dataset to be tested on, as previous work (Garforth and Webb, 2019) has shown that SLAM has difficulty tracking forest videos, and in order to test a loop closure we need continuous tracking between the first and second visit to the loop location. This has not been achieved on Hillwood, and SFU contains only challenging 180° rotated loop closures. Our new “Forest Loop” dataset was gathered specifically to provide both a smooth motion to aid in tracking and a closed circuit to test our loop closure system on real robot data.

## 4. Results

### 4.1. Classifying Forests

As can be seen in Table 1, VGG-16 achieves reasonable classification of forests on all real datasets. More than half of the misclassifications of SFU Forest dry are as “field road” which is reasonable given that some open track still exists in the dataset. Hillwood AR is primarily misclassified as “trench,” “driveway,” or “yard.” The reason for these is less obvious, but this dataset was recorded at a time of year when a lot of leaves were on the ground, a feature that was perhaps also present in some of the original training data for “driveway” and “yard.”

**Table 1:** Percentage of correct classification matches achieved by NetVLAD when using a different camera from the stereo pair for the query vs. database dataset.

**Dataset**	**Forest**	**Other common labels**
Hillwood Bebop	99.10%	Orchard (0.28%), trench (0.20%)
SFU Forest Dry	86.31%	Field road (9.64%), corn field (1.18%)
Hillwood AR	80.00%	Trench (6.21%), driveway (3.41%), yard (2.51%)
Forest Loop	77.69%	Trench (14.81%), yard (2.21%), landfill (1.66%)
Unreal	60.95%	Fishpond (15.84%), pond (5.71%), aquarium (1.87%)

The simulated forest data is classified significantly less accurately than real data, with the most common non-forest labels being pond, fishpond and aquarium (which account for 23.63% of classifications between them). The artificial lighting conditions seem the most likely explanation for this, which is not unsurprising given the results of Garforth and Webb (2019) and furthers their warning about using simulated data to test algorithms meant to work in the real world.

Aside from in simulation, initial results show that the network underlying NetVLAD is able to learn features specific to forests, which reassures us that full NetVLAD has the potential for describing places within forests.

### 4.2. Place Recognition Against Baseline

Table 2 shows the rates of correct matches achieved by NetVLAD between pairs of routes from the SFU dataset under various environmental conditions. These are presented alongside the results for other place recognition systems from Lowry and Andreasson (2018) for the same pairings, where available.

**Table 2:** Percentage of correct place matches achieved by different place recognition algorithms on pairs of routes under different environmental conditions from the SFU dataset.

**Datasets**	**BOW**	**FAB-MAP**	**VLAD**	**NetVLAD Full**	**NetVLAD Forest**
Dry-Dry	–	–	–	97.3	93.9
Dry-Wet	12.6	18.4	28.5	37.0	50.0
Dry-Dusk	15.1	22.6	32.2	47.3	53.3
Dry-Night	–	–	–	9.59	30.0

*Results for the first three algorithms are from the work of Lowry and Andreasson (2018). The fourth column reports on NetVLAD, and the final covers the same algorithm but reports only for the final canopy-covered section of the dataset*.

The rates of correct matches in Table 2 show NetVLAD achieves a substantial increase over VLAD features, as well as BoW and FAB-MAP, in both previously tested forest setups. Notably we have taken the best result across descriptor sizes for BoW, FAB-MAP, and VLAD, so in most cases our 128 dimension NetVLAD descriptor would also use less memory. This makes a strong case for using it in the loop closure settings where those other descriptors are typically used.

Somewhat surprisingly, NetVLAD also performs better on the canopy-covered sections of forest at the end of the SFU datasets than it does on the track as a whole. The track at the start of the SFU videos must be difficult to distinguish, likely due to most frames consisting solely of a road and surrounding treeline. To the eye these images are very similar, as noted in Garforth and Webb (2019). Under the canopy, however, the more complex skyline has been shown to be useful in navigation before (Stone et al., 2016), so we posit that this is part of what the network is using to achieve its improved performance.

Under canopy cover NetVLAD also shows resistance to changing environmental conditions, achieving performance on the difficult dry-night pairing that matches VLAD's performance on the two easier pairings. The skyline is not visible in the night time data, so the performance here implies that this isn't the only useful feature being learned.

### 4.3. Evaluation Without Ground Truth

Table 3 shows the match percentages when comparing two datasets produced by subsampling from a single video. We varied the number of frames offset between the two subvideos and as would be expected we find the performance to be better when this is closer to 0 or to the step size, as this means frames recorded closer in time exist in the two subvideos. Even at the worst case for either dataset (58.45% for Hillwood AR or 65.0% for Bebop) performance does not diminish too much, suggesting a resistance to translations in the NetVLAD descriptors.

**Table 3:** Percentage of correct place matches when sub-sampling a single video to produce database and query sets.

		**Offset (as fraction of step)**				
**Dataset**	**Step size**	**1/6**	**1/3**	**1/2**	**2/3**	**5/6**
Hillwood AR	36	64.34	63.64	60.14	58.45	64.09
Hillwood Bebop	90	75.83	65.0	69.17	71.43	71.43

*Step size (in frames) indicates rate at which frames are sampled and is set to the equivalent of 3 s. Offset indicates distance between frames assigned to different sets*.

### 4.4. Loop Closure

**Figure 2:**
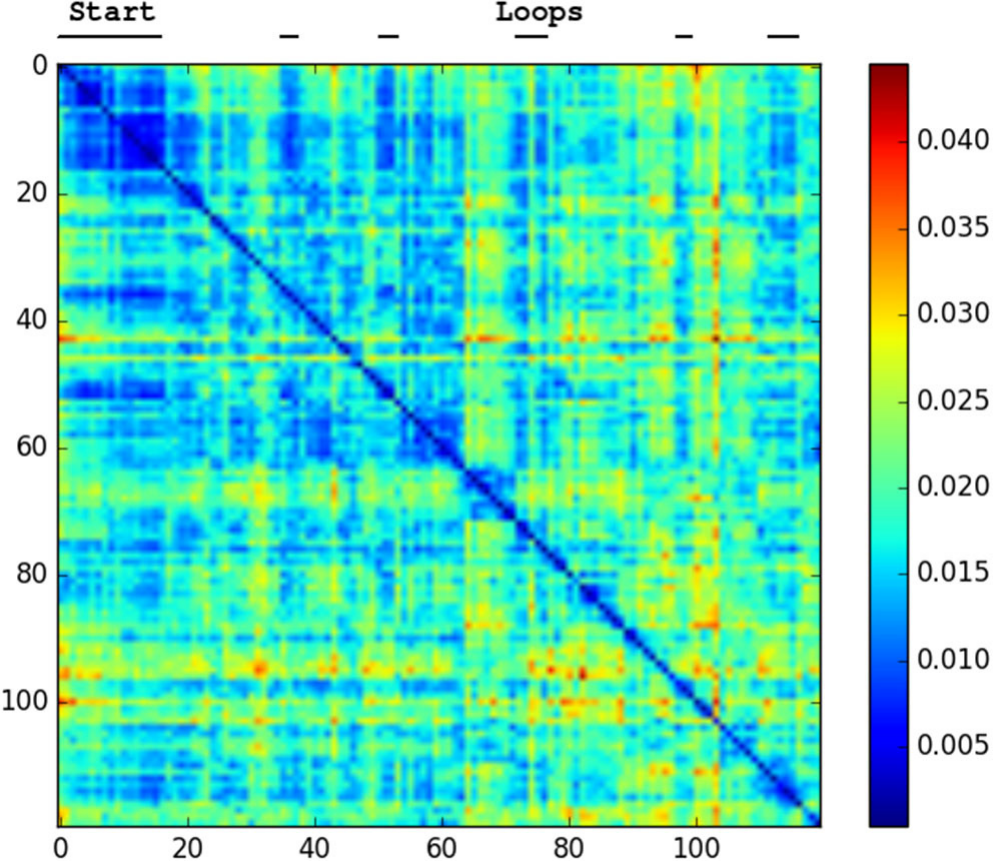
Confusion matrix showing the squared euclidean distance between frames of Hillwood Bebop. Note multiple loops returning to the start as shorter distance patches.

**Figure 3:**
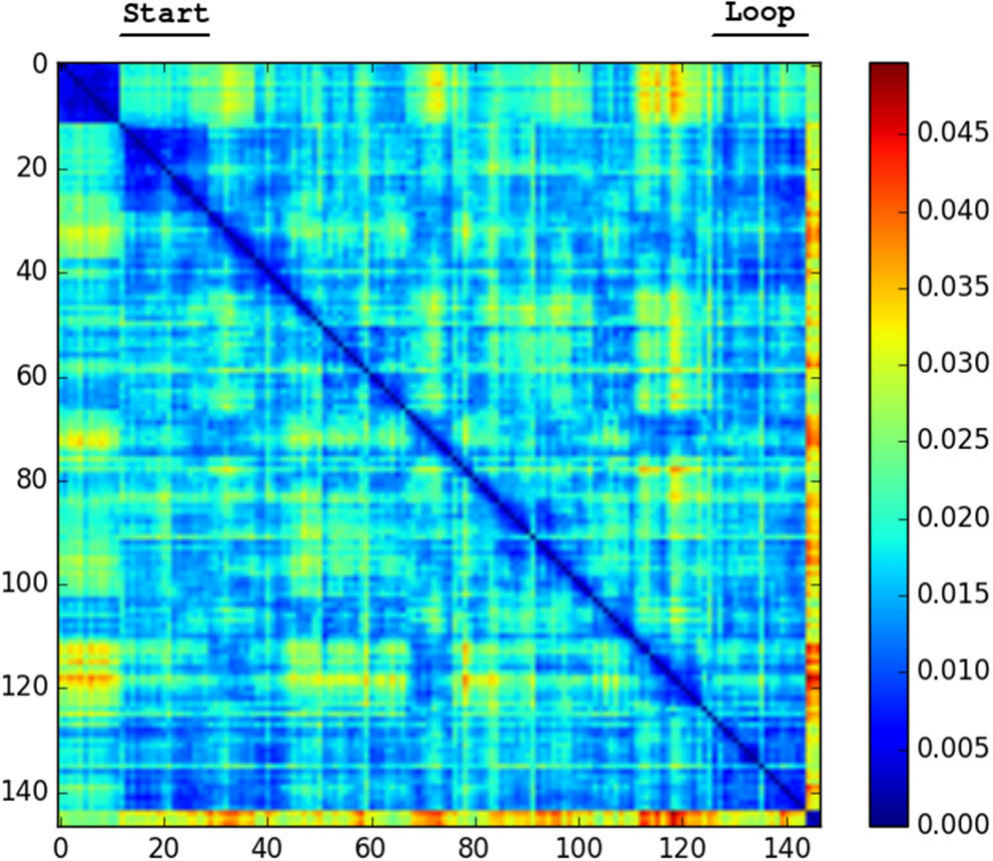
Confusion matrix showing the squared euclidean distance between frames of Forest Loop. Loop closure at the end of the video noticeable as shorter distance patch.

**Figure 4:**
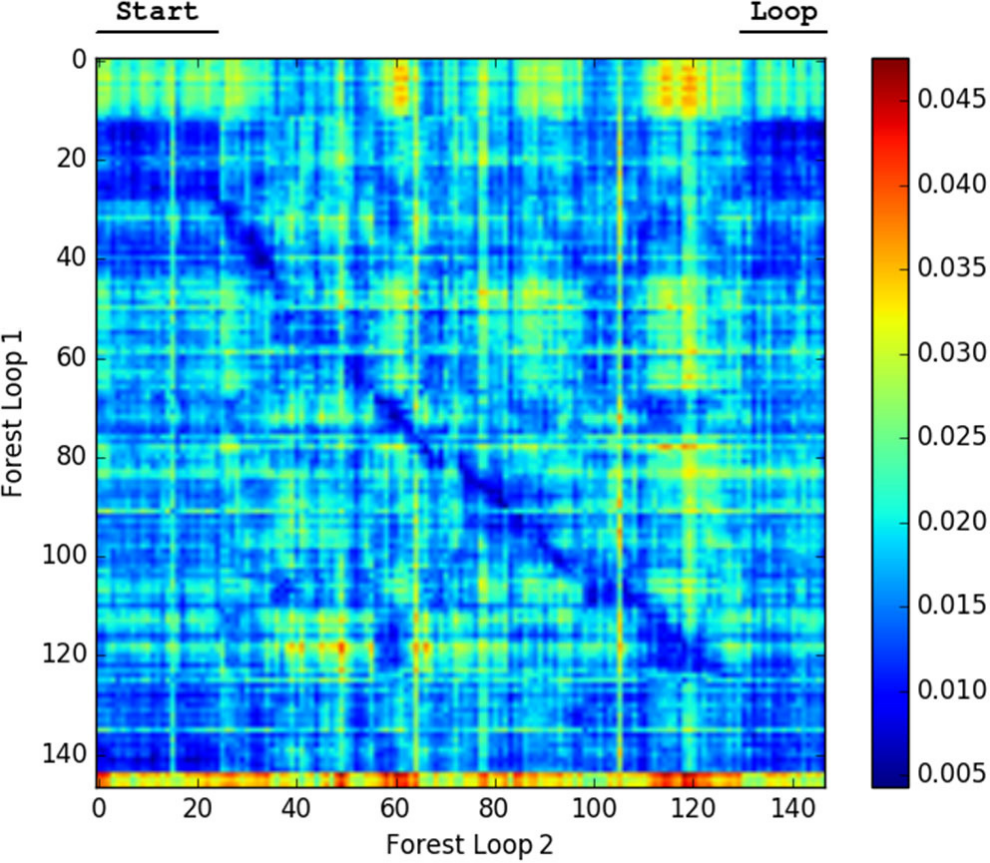
Confusion matrix showing the squared euclidean distance between frames of Forest Loop on two different passes. The matching of the route near the diagonal and the loop are both visible.

We generated confusion plots of distances between pairs of NetVLAD descriptions for videos from robots traversing forest terrain. We can make out columns of blue (low distance) in Figures 2, 3 at the indicated points where the path loops back to previous visited locations, showing that the descriptor comparison is able to successfully detect the similarity of these forest locations. There are some other patches at points we had not identified as full loops, but it is worth noting that in forested conditions especially these “places” are not straightforward to define, and parts of one scene may indeed be visible in a part of another view from another part of the route, so some noise is expected. Occasional incorrect matches would be dealt with in a SLAM application through filtering and feature correspondence checks. Additionally, as a baseline check, we compared two different traversals of the same Forest Loop route in Figure 4 and can see a clear path of high similarity near the diagonal between the two videos.

**Figure 5:**
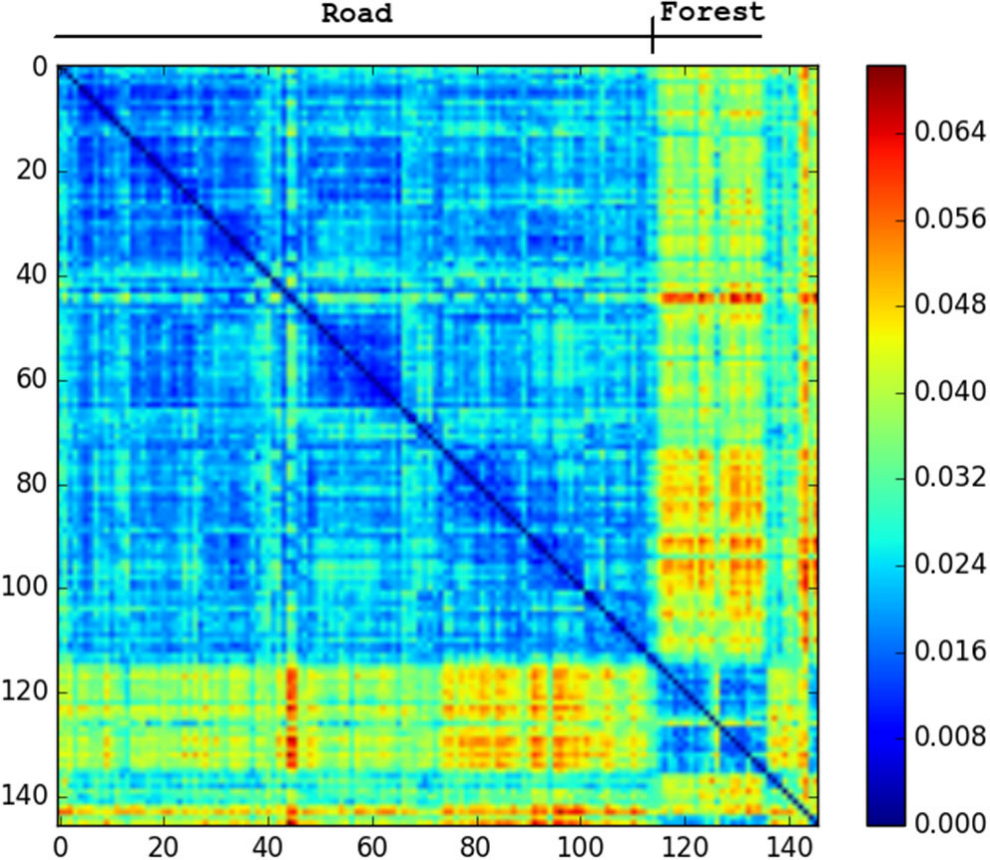
Confusion matrix showing the squared euclidean distance between frames of SFU dry. Road and forest sections very different from each other. No loop matches.

The only repeated traversals of the same path in the SFU video occur while facing in opposite directions. These are challenging conditions for any place recognition, so this video is test of how far NetVLAD can be pushed. Figure 5 shows that the descriptor comparison makes a clear distinction between forest and road sections of the SFU video, but within these sections most places seem equally similar to each other. We would not expect to be able to close the loop in this dataset with NetVLAD.

**Figure 6:**
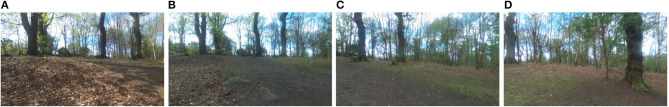
Place matches achieved on Hillwood Bebop dataset demonstrate robustness to rotation [view is shifting right from **(A)** to **(D)**], illumination changes [sun passes behind cloud between **(A)** and **(B)**] and onset of previously unseen occluding objects [large tree comes into view in foreground between **(C)** and **(D)**].

If we look at sample frames from the regions NetVLAD indicates are similar in Figure 6, we can see that the network is producing low distance scores in spite of the presence of potentially confusing factors, such as a rotated camera view, a prominent object (tree) not previously visible, and a change in lighting affecting the apparent texture of the ground.

**Figure 7:**
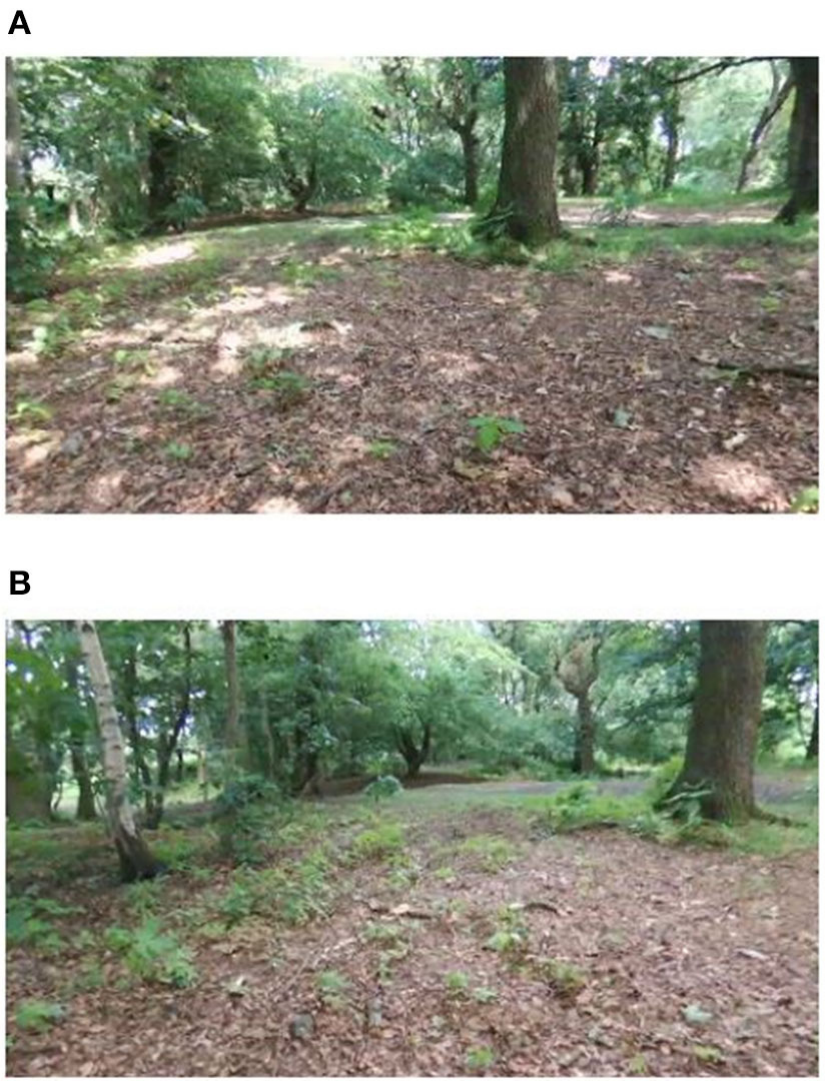
Example correct place match proposed by NetVLAD between the beginning **(A)** and end **(B)** of Forest Loop.

**Figure 8:**
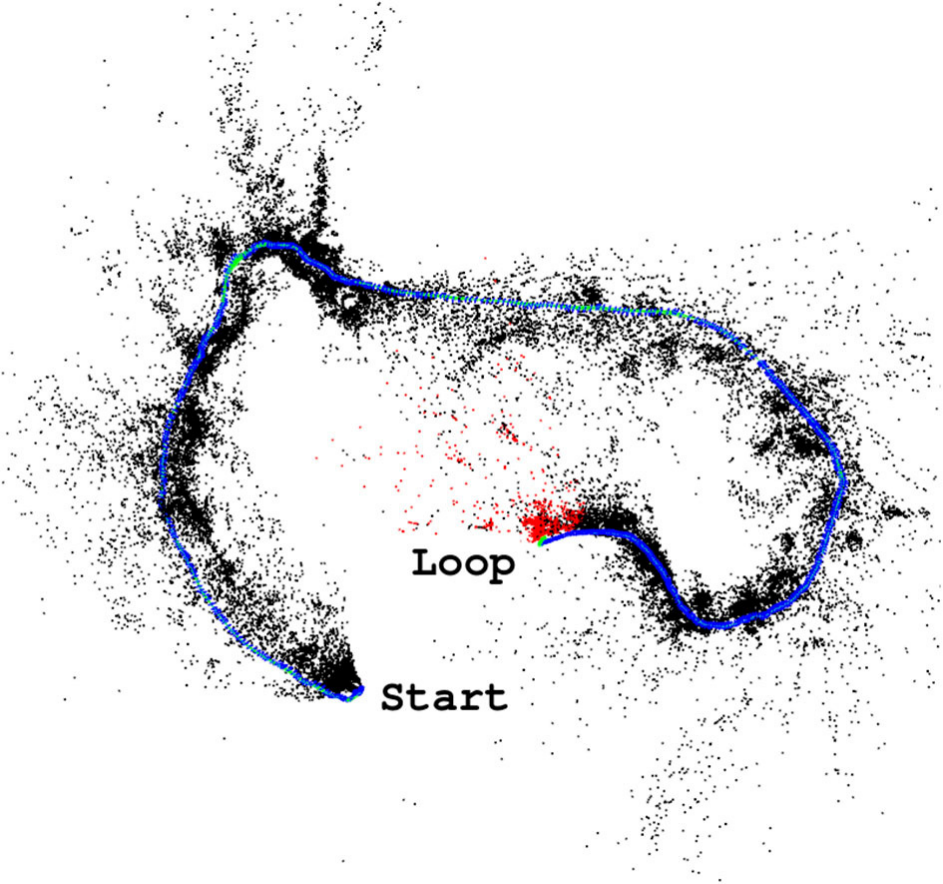
ORBSLAM2 (Mur-Artal et al., 2015) successfully tracks the Forest Loop video, but inevitable drift results in the start and end locations not connecting as they should.

The results so far demonstrate this network's potential for use as a loop closure mechanism for SLAM in forests. Given this, our final experiment was the integration of NetVLAD descriptors in ORBSLAM2 as a loop closure proposal mechanism. Unfortunately, even though the system proposed loop closures at appropriate frames (such as those shown in Figure 7), the alignment of the point clouds required for the global map optimization always failed to align the features from the two matched frames. The resulting map can be seen in Figure 8 with the loop still open.

The likely cause of these difficulties is the very highly textured scenes providing an abundance of potential features, or the notable change in lighting that occurs even in the few minutes between the images in Figure 7, such that repeated visits to the same location do not have a large enough overlap in extracted features to find an alignment. This was a risk, given the previously reported poor performance of SLAM systems in these environments in Garforth and Webb (2019), and shows that more work must be done on the frame to frame tracking part of the mapping process before a full working forest SLAM system can be produced.

### 4.5. Scene Statistics

As we are reporting work with a new forest dataset, we present here the scene statistics (as described in Garforth and Webb, 2019) of that data, compared to the previously existing datasets. This data was also recorded using the Bebop Drone, so it notably has the same camera properties as “Hillwood_Bebop.”

**Figure 9:**
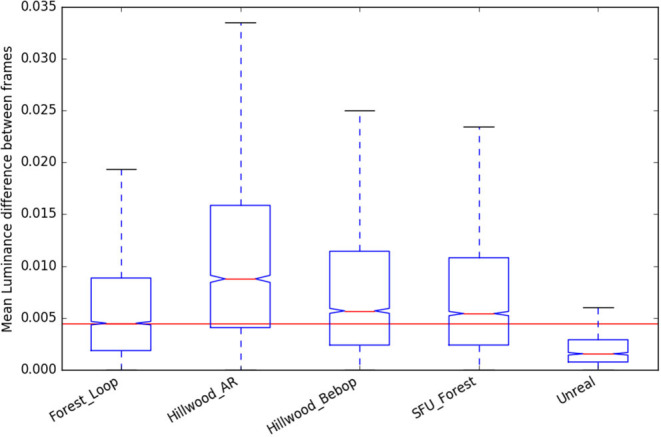
Luminance change statistics of the new “Forest Loop” dataset, compared to existing.

**Figure 10:**
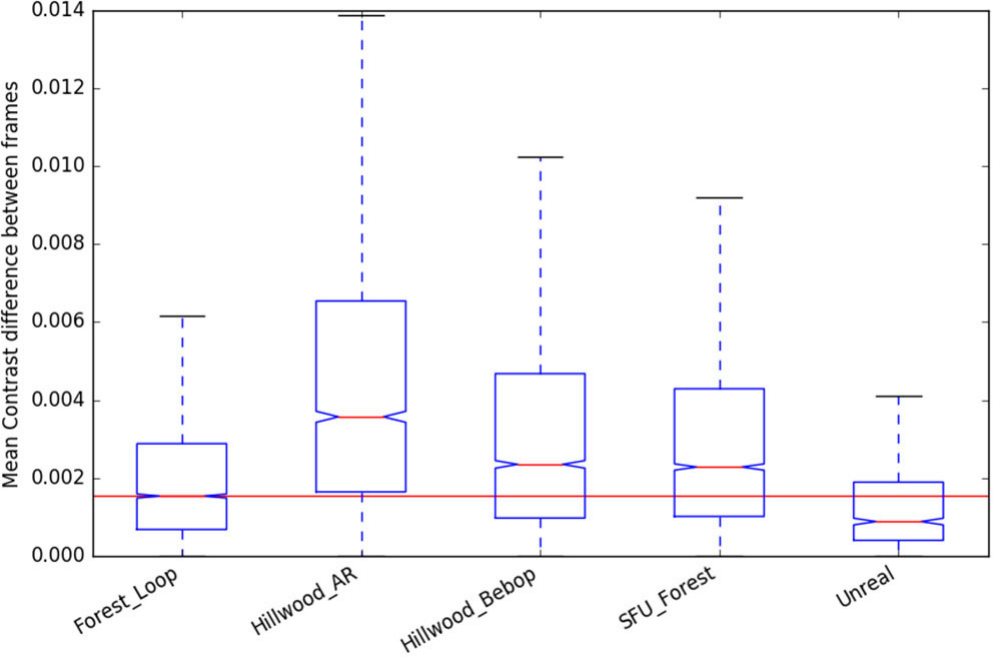
Contrast change statistics of the new “Forest Loop” dataset, compared to existing.

**Figure 11:**
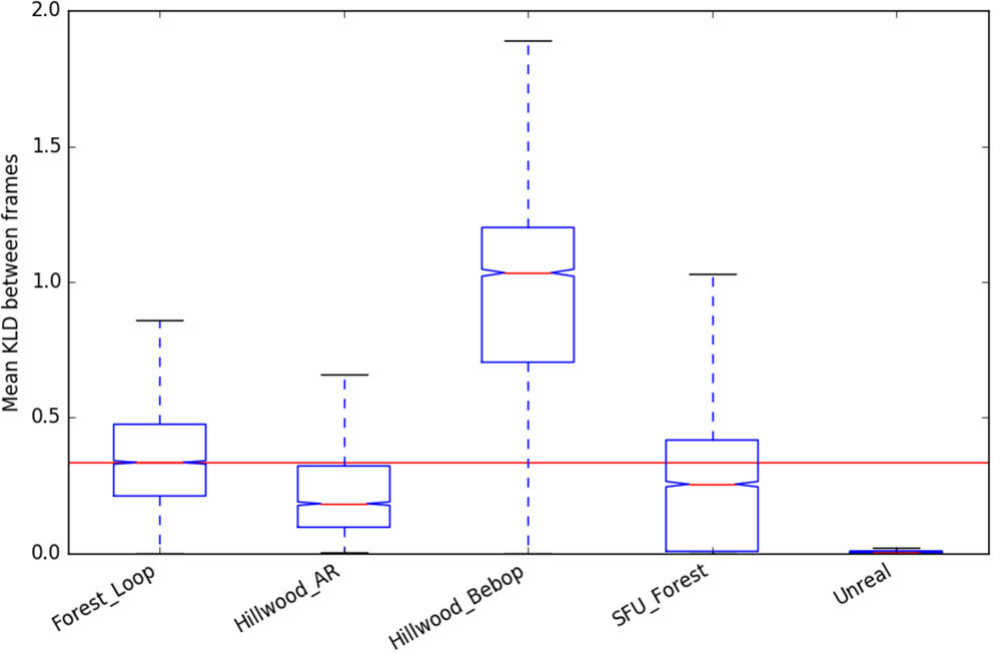
KL Divergence statistics (used as a representation of in-scene motion) of the new “Forest Loop” dataset, compared to existing.

We can see that “Forest_Loop” is less variable in its lighting (Figures 9, 10) than other forest datasets, while its in scene motion (as measured by KL divergence in Figure 11) is indistinguishable. No specific care was taken to avoid areas based on lighting, but this data was recorded at a careful pace, keeping a good distance away from foliage in order to improve matching chances. Easier to track data forest data was our aim, and these statistics would lead us to believe it has been at least partially achieved.

## 5. Conclusion and Future Work

In this paper we presented an investigation into the use of place recognition network NetVLAD for loop closure in challenging forest mapping scenarios. Even though NetVLAD itself has not been trained to recognize places in forests, we showed that its underlying VGG-16 network does categorize a variety forest datasets correctly. We then demonstrated that NetVLAD performs better than state of the art loop closure approaches in this difficult environment, robust to the presence of changing lighting, time of day, weather conditions, rotation, and translation. Our results marked NetVLAD as a candidate for proposing loop closures for SLAM, and we put together a test system as well as gathering new data to confirm. We found that NetVLAD reliably proposed loop closures to ORBSLAM, but that the frame to frame ORB feature matching of the SLAM system was not able to integrate these into the map. This is a useful warning for anyone attempting SLAM in forests, and supports feature matching as one avenue for further research.

A number of other improvements can be made to our test integration with SLAM to produce a system ready for deployment. Given ORBSLAM's difficulty aligning feature points at proposed loop closures, we would propose investigating a feature-less “direct” SLAM method like DSO instead. Once a pairing with good performance is found, we would replace our offline NetVLAD descriptor calculation with one that runs in real-time. Finally, to improve scalability as the map gets larger we would consider a hierarchical graph and sequence based search method, like that described in Vysotska and Stachniss (2019).

## Data Availability Statement

The SFU dataset can be found at http://autonomy.cs.sfu.ca/sfu-mountain-dataset/. The rest of the data supporting the conclusions of this article will be made available by the authors, without undue reservation, to any qualified researcher.

## Author Contributions

The work for this paper was undertaken by JG. Supervision and proof reading were provided by BW. All authors contributed to the article and approved the submitted version.

## Conflict of Interest

The authors declare that the research was conducted in the absence of any commercial or financial relationships that could be construed as a potential conflict of interest.
